# 

**Published:** 1890-03-01

**Authors:** 


PRICE ONE PENNY.

No. 179, Vol. VII.—March 1st, 1890.

[Registered at the General Post Office as a Newspaper for transmission in the United Kingdom and Abroad.

WITH EXTRA NURSING SUPPLEMENT.
Per Annum, 6s. 6d.; post free.
Monthly Parts, 6d.; post free 8d.
Ditto, per Annum, 8s., post free.

